# Comparison between left bundle branch area pacing and right ventricular pacing: ventricular electromechanical synchrony and risk of atrial high-rate episodes

**DOI:** 10.3389/fcvm.2024.1267076

**Published:** 2024-04-25

**Authors:** Wang-Yang Yang, Bei-Bing Di, Hui Peng, Zhi-Jun Sun

**Affiliations:** Department of Cardiology, Cardiovascular Center, Beijing Friendship Hospital, Capital Medical University, Beijing, China

**Keywords:** left bundle branch area pacing, right ventricular pacing, two-dimensional speckle tracking echocardiography, synchrony, atrial high-rate episodes

## Abstract

**Background:**

The electromechanical dyssynchrony associated with right ventricular pacing (RVP) has been found to have adverse impact on clinical outcomes. Several studies have shown that left bundle branch area pacing (LBBAP) has superior pacing parameters compared with RVP. We aimed to assess the difference in ventricular electromechanical synchrony and investigate the risk of atrial high-rate episodes (AHREs) in patients with LBBAP and RVP.

**Methods:**

We consecutively identified 40 patients with atrioventricular block and no prior atrial fibrillation. They were divided according to the ventricular pacing sites: the LBBAP group and the RVP group (including the right ventricular apical pacing (RVA) group and the right side ventricular septal pacing (RVS) group). Evaluation of ventricular electromechanical synchrony was implemented using electrocardiogram and two-dimensional speckle tracking echocardiography (2D-STE). AHRE was defined as event with an atrial frequency of ≥176 bpm lasting for ≥6 min recorded by pacemakers during follow-up.

**Results:**

The paced QRS duration of the LBBAP group was significantly shorter than that of the other two groups: LBBAP 113.56 ± 9.66 ms vs. RVA 164.73 ± 14.49 ms, *p* < 0.001; LBBAP 113.56 ± 9.66 ms vs. RVS 148.23 ± 17.3 ms, *p* < 0.001. The LBBAP group showed shorter maximum difference (TDmax), and standard deviation (SD) of the time to peak systolic strain among the 18 left ventricular segments, and time of septal-to-posterior wall motion delay (SPWMD) compared with the RVA group (TDmax, 87.56 ± 56.01 ms vs. 189.85 ± 91.88 ms, *p* = 0.001; SD, 25.40 ± 14.61 ms vs. 67.13 ± 27.40 ms, *p* < 0.001; SPWMD, 28.75 ± 21.89 ms vs. 99.09 ± 46.56 ms, *p* < 0.001) and the RVS group (TDmax, 87.56 ± 56.01 ms vs. 156.46 ± 55.54 ms, *p* = 0.003; SD, 25.40 ± 14.61 ms vs. 49.02 ± 17.85 ms, *p* = 0.001; SPWMD, 28.75 ± 21.89 ms vs. 91.54 ± 26.67 ms, *p* < 0.001). The interventricular mechanical delay (IVMD) was shorter in the LBBAP group compared with the RVA group (−5.38 ± 9.31 ms vs. 44.82 ± 16.42 ms, *p* < 0.001) and the RVS group (−5.38 ± 9.31 ms vs. 25.31 ± 21.36 ms, *p* < 0.001). Comparing the RVA group and the RVS group, the paced QRS duration and IVMD were significantly shorter in the RVS group (QRS duration, 164.73 ± 14.49 ms vs. 148.23 ± 17.3 ms, *p* = 0.02; IVMD, 44.82 ± 16.42 ms vs. 25.31 ± 21.36 ms, *p* = 0.022). During follow-up, 2/16 (12.5%) LBBAP patients, 4/11 (36.4%) RVA patients, and 8/13 (61.5%) RVS patients had recorded novel AHREs. LBBAP was proven to be independently associated with decreased risk of AHREs than RVP (log-rank *p* = 0.043).

**Conclusion:**

LBBAP generates narrower paced QRS and better intro-left ventricular and biventricular contraction synchronization compared with traditional RVP. LBBAP was associated with a decreased risk of AHREs compared with RVP.

## Highlights

• This study demonstrated differences in electromechanical synchrony between LBBAP and RVP using non-invasive clinical examinations, including electrocardiogram and two-dimensional speckle tracking echocardiography (2D-STE).
• LBBAP was found to be associated with a more physiological ventricular activation, as evidenced by a narrower paced QRS duration and better intro-left ventricular and bi-ventricular contraction synchronization compared to RVP.
• LBBAP was proven to be associated with a decreased risk of AHREs compared with RVP. The mechanism of ventricular pacing associated AHREs is unclear, but the left atrial dysfunction caused by left ventricular dyssynchrony may be an important explanation.

## Introduction

More than half a century of clinical practice has demonstrated the effectiveness of traditional right ventricular pacing (RVP) in the treatment of bradycardia. Generally, the most commonly used RVP consists of right ventricular apical pacing (RVA) and right side ventricular septal pacing (RVS). However, the electromechanical dyssynchrony associated with RVP has been found to have an adverse impact on clinical outcomes. It is reported that RVP is associated with an increased risk of heart failure and cardiomyopathy (1, 2). Recent studies have also found that RVP may increase the risk of new-onset atrial arrhythmias (3–6). Alternative pacing sites that generate more physiological electromechanical activation of the ventricle may lead to more clinical benefit. Currently, physiological conduction system pacing is recommended for patients with high pacing proportions to reduce the risk of adverse prognosis (7). The left bundle branch area pacing (LBBAP) can selectively or non-selectively capture the left bundle branch (LBB), making the left ventricular depolarization procedure closer to physiological conduction (8). Several studies have shown that LBBAP has superior pacing parameters compared with RVP (7, 9). This alternative pacing site appears to be very promising, but data on this new technique is still scarce. Studies evaluating LBBAP in ventricular electromechanical synchrony and its potential influence on atrial arrhythmia are needed. Two-dimensional speckle tracking echocardiography (2D-STE) can provide a comprehensive assessment of ventricular mechanical synchrony by analyzing parameters of motion and deformation of the 18 ventricular wall segments in all three axes. QRS duration in the electrocardiogram can represent ventricular electrical synchrony. Atrial high-rate episode (AHRE), a pacemaker-recorded atrial tachyarrhythmia (10), is considered a manifestation of atrial electrical remodeling, characteristic of atrial cardiomyopathy. In this study, we aimed to evaluate differences in ventricular electromechanical synchrony and investigate the risk of AHREs in patients with LBBAP, RVA, and RVS, respectively.

## Methods

### Study population

In this retrospective study, we consecutively identified patients with atrioventricular block (AVB) referred to Beijing Friendship Hospital for permanent dual-chamber pacemaker implantation (7) between December 2017 and June 2022. All patients fulfilled the following inclusion criteria: (i) underwent dual-chamber pacemaker implantation; (ii) underwent two-dimensional speckle tracking echocardiography (2D-STE) a week after pacemaker implantation; (iii) had stable pacemaker electrical parameters (no need for reprogramming). The exclusion criteria were as follows: (i) prior history of atrial fibrillation (AF) or underwent AF catheter or surgical ablations, moderate to severe mitral or aortic stenosis, moderate to severe mitral regurgitation, and cardiomyopathy; (ii) left ventricular ejection fraction less than 40%; (iii) ventricular pacing percentage less than 70%; (iv) follow-up time less than 6 months. The protocol of our study has been approved by the Institutional Review Board of Beijing Friendship Hospital affiliated to Capital Medical University and is in accordance with the Declaration of Helsinki. All patients gave informed consent.

Patients were divided according to the ventricular pacing sites: the LBBAP group and the RVP group (including the RVA group and the RVS group). LBBAP was achieved if the pacing morphology of the QRS complex showed an RBBB pattern and fulfilled either of the following criteria (11–13): (i) confirmation of recording LBB potential; (ii) the stimulus to left ventricular activation time (Stim-LVAT) shortening abruptly with increasing output or remaining shortest and constant (<90 ms) at either low or high outputs. The initial pacing setting utilized in this study was bipolar pacing with output of 3.5 V/0.42 ms (automatic adjustment of pacing output 100 days after the procedure), DDD mode with a base rate of 60 bpm, and the initial atrioventricular (AV) delay was set at 150 ms-sensed AV delay and 180 ms-paced AV delay.

### Evaluation of electrical and mechanical synchrony

For all patients, 12-lead surface electrocardiograms (ECGs, 25 mm/s paper speed with a gain of 10 mm/mV) were performed before and after the pacemaker implantation procedure to confirm that the ventricular activation was pacemaker-driven. The native and paced duration of the QRS complex was measured from the end of the PR interval to the end of the S-wave, primarily from leads II, III, and aVF. This measurement aimed to reflect the electrical synchrony of ventricular depolarization.

**Figure 1:**
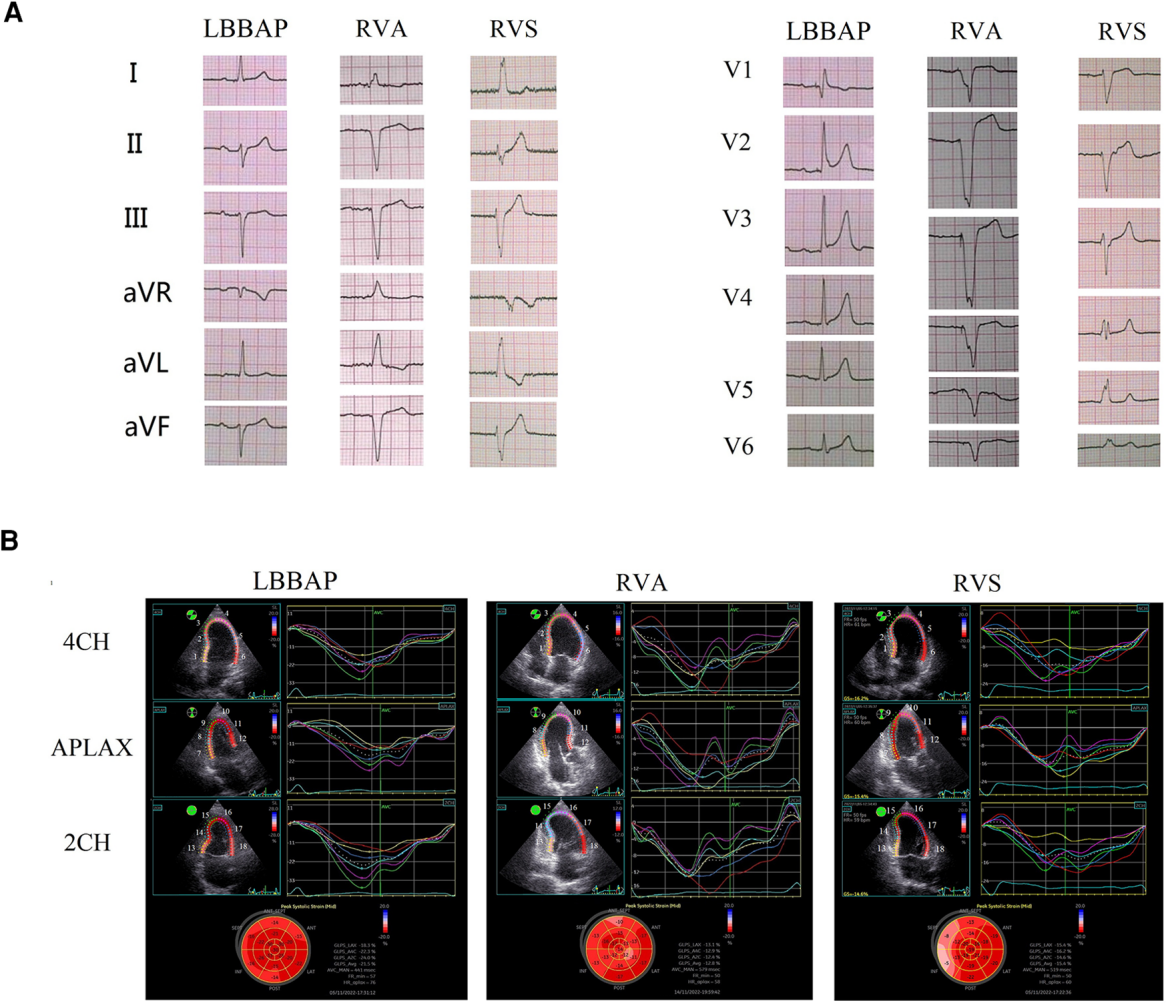
Electromechanical synchrony evaluation in patients with LBBAP vs RVA vs RVS. (**A**) paced surface 12-lead electrocardiograms for LBBAP vs RVA vs RVS; (**B**) time-systolic strain curves of the four-chamber view (4CH), apical long-axis view (APLAX), and two-chamber view (2CH), and the longitudinal strain bull's eye plot for LBBAP vs RVA vs RVS. The serial number of 18 left ventricular wall segments in the figure: 4CH view: 1, Basal-septal; 2, Mid-septal; 3, Apical-septal; 4, Apical-lateral; 5, Mid-lateral; 6, Basal-lateral. APLAX view: 7, Basal-posterior; 8, Mid-posterior; 9, Apical-posterior; 10, Apical-anteroseptal; 11, Mid-anteroseptal; 12, Basal-anteroseptal. 2CH view: 13, Basal-inferior; 14, Mid-inferior; 15, Apical-inferior; 16, Apical-anterior; 17, Mid-anterior; 18, Basal-anterior.

Evaluation of ventricular mechanical synchrony was implemented by 2D-STE after the pacemaker implantation (14, 15). The images and heart rate of four cardiac cycles were continuously collected and evaluated, and the standard four-chamber (4CH) view, apical long-axis (APLAX) view, and two-chamber (2CH) view with clear display were selected for measurement. The times to 2D longitudinal peak systolic strain of 18 segments were recorded from these three standard views (as shown in Figure 1B). Characteristics representing intraventricular synchrony included the maximum difference (TDmax) and the standard deviation (SD) of the time to peak systolic strain among the 18 left ventricular segments, as well as the time of septal-to-posterior wall motion delay (SPWMD). The time difference between the left and right ventricular pre-ejection periods is called the interventricular mechanical delay (IVMD), which can reflect the interventricular synchrony. In measurement, IVMD refers to the time from the beginning of the QRS complex to the beginning of aortic or pulmonary valve blood flow. The 2D-STE was performed by two senior sonographers after the pacemaker implantation. The VVIq ultrasound (GE Company, USA) with S-5 transducers was used. The offline software TOMTEC (Tom-Tec Imaging Systems, Unterschleissheim, Germany) was used to calculate parameters representing the synchrony status.

### Baseline information and follow-up

All those data were validated through manual review. All patients had regular follow-up visits at the device outpatient clinic at 1, 6, and 12 months, and annually afterward post pacemaker implantation. Pacing parameters (sensing amplitude, stimulation threshold, and impedance), heart rhythm events, and the percentage of ventricular pacing were routinely checked. The follow-up period was counted from the initial procedure day to the end of the observational period (the censoring date of December 31, 2022), or the date of the first AHRE event, or loss of follow-up, whichever came first.

AHREs were defined as events with an atrial frequency of ≥176 bpm lasting for ≥6 minutes recorded by pacemakers during follow-up. The intracardiac electrograms of all AHREs were manually checked. The controversial events were adjudicated by at least two junior investigators by reviewing the patient's medical records separately.

### Statistical analysis

Continuous variables were presented as means ± standard deviations, or medians (quartile1-quartile3) and compared by *T*-tests or Mann-Whitney *U* tests, respectively. Categorical variables were presented as numbers (percentages) and compared by the Chi-squared (*χ*^2^) test. The Kaplan-Meier curve was used to directly display the cumulative incidence of AHREs in the LBBAP, RVA, and RVS groups, with a log-rank test comparing the difference. All the analyses were performed using SPSS Statistics version 22.0 (IBM). A two-sided *P*-value < 0.05 was considered statistically significant.

## Results

**Figure 2:**
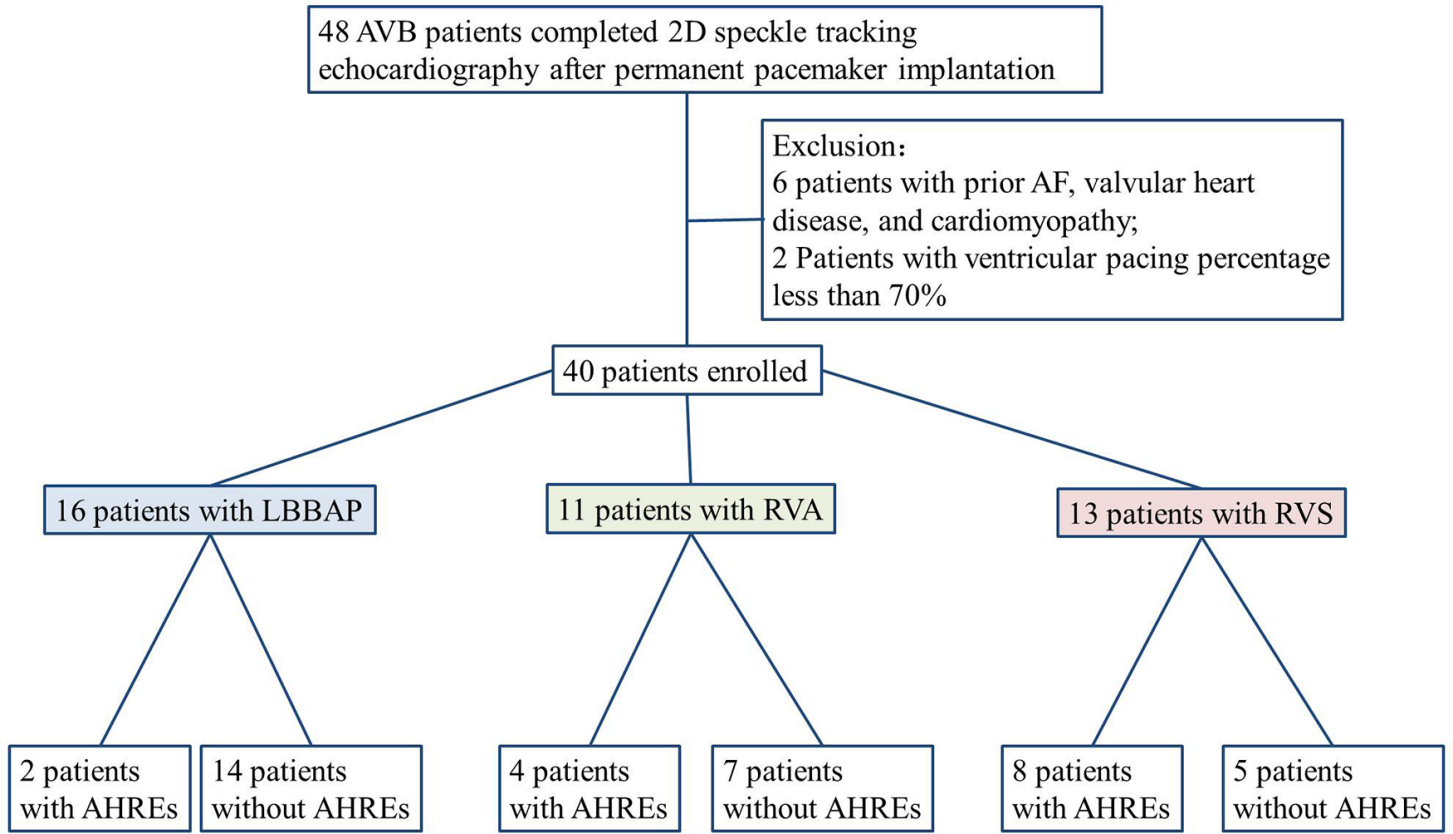
Flowchart of enrolled patients according to inclusion and exclusion criteria. AF, atrial fibrillation; LBBAP, left bundle branch area pacing; RVA, right ventricular apical; RVS, right side of ventricular septal; AHREs, atrial high-rate episodes.

A total of 48 AVB patients were examined with 2D-STE after permanent dual-chamber pacemaker implantation between December 2017 and June 2022. Six patients with previously diagnosed AF and two patients with ventricular pacing percentages less than 70% were excluded. Finally, 16 LBBAP, 11 RVA, and 13 RVS patients were analyzed (Figure 2). Among these 16 LBBAP patients, 8 exhibited recorded LBB potentials. The types of AVB in the three groups are: LBBAP group, 4/16 (25%) patients with III° AVB, 11/16 (68.75%) patients with II° AVB (Mobitz type II), and 1/16 (6.25%) patient with triple branch block (first degree AVB, RBBB, left anterior fascicular block); RVA group, 7/11 (63.64%) patients with III° AVB and 4/11 (36.36%) patients with II° AVB (Mobitz type II); RVS group, 2/13 (15.38%) patients with intermittent high-degree AVB, 5/13 (38.46%) patients with III° AVB, and 6/13 (46.15%) patients with II° AVB (Mobitz type II). The ventricular pacing percentage of all patients was above 85% during follow-up.

There was no significant difference in baseline characteristics compared pairwise among the LBBAP vs. RVA vs. RVS groups (Table 1). The left ventricular ejection fraction (LVEF) at baseline in the LBBAP vs. RVA vs. RVS groups were 66.81 ± 9.38% vs. 62.13 ± 8.93% vs. 65.04 ± 2.87%, respectively. The native QRS duration was 112.63 ± 21.23 ms in the LBBAP group, 108.09 ± 15.3 ms in the RVA group, and 110.69 ± 28.73 ms in the RVS group. The baseline median NT-proBNP was 144 (74, 392.75) ng/L in the LBBAP group, 1,582 (292, 3,335) ng/L in the RVA group, and 279 (184.5, 1,736.5) ng/L in the RVS group.

**Table 1:** Baseline characteristics.

	LBBAP (N1 = 16)	RVA (N2 = 11)	RVS (N3 = 13)	*P* value (LBBAP vs RVA)	*P* value (LBBAP vs RVS)	*P* value (RVA vs RVS)
Age, years	68.19 ± 14.77	67.64 ± 15.49	75.08 ± 9.01	0.926	0.153	0.157
Sex (male), *n* (%)	5 (31.25)	5 (45.45)	7 (53.45)	0.687	0.274	1.000
Hypertension, *n* (%)	11 (68.75)	8 (72.73)	12 (92.31)	1.000	0.183	0.3
Diabetes mellitus, *n* (%)	10 (62.5)	5 (45.45)	5 (38.46)	0.452	0.272	1.000
Coronary artery disease, *n* (%)	4 (25)	4 (36.36)	5 (38.46)	0.675	0.688	1.000
NT-proBNP, ng/L	144 (74, 392.75)	1,582 (292, 3,335)	279 (184.5, 1,736.5)	0.122	0.276	0.203
Electrocardiography						
QRS duration, ms	112.63 ± 21.23	108.09 ± 15.3	110.69 ± 28.73	0.55	0.837	0.781
Right bundle branch block, *n* (%)	7 (43.75)	4 (36.36)	6 (46.15)	1.000	1.000	0.697
Echocardiography						
LAD, mm	37.61 ± 4.23	39.76 ± 6.59	40.1 ± 5.94	0.310	0.199	0.897
LVEDD, mm	49.46 ± 4.10	52.06 ± 8.61	51.59 ± 5.34	0.206	0.484	0.321
LVEF, %	67.31 ± 7.02	62.39 ± 8.89	64.92 ± 3.37	0.368	0.236	0.869

LBBAP, left bundle branch area pacing; RVA, right ventricular apical pacing; RVS, right side of ventricular septal pacing; RBBB, right bundle branch block; LAD, left atrium anteroposterior diameter; LVEF, left ventricular ejection fraction; LVEDD, left ventricular end-diastolic diameter.

The median follow-up time of the LBBAP, RVA, and RVS groups were 1,136 (729, 1,312) vs. 858 (701, 1,263) vs. 902 (339, 1,314) days, respectively. No one was lost of follow-up. None of the patients experienced infection, hematoma, or lead complications after pacemaker implantation or during follow-up. At the last recorded outpatient follow-up, regular echocardiographic results of all 40 patients showed that the LVEF was within the normal range, and there was no significant difference between the three groups (LBBAP group 66.19 ± 6.29% vs. RVA group 62.36 ± 5.24% vs. RVS group 64.08 ± 4.63%). During follow-up, the NT-proBNP values also showed no significant difference between the three groups, LBBAP group 133 (66, 265.75) ng/L vs. RVA group 219 (66, 1,361) ng/L vs. RVS group 678 (66, 1,298.5) ng/L.

### Electromechanical synchrony

There was no significant difference in the baseline QRS duration of these three groups (Table 1). As a routine preoperative examination, conventional echocardiography was used to exclude patients with abnormal ventricular wall motion. Given that the patients with AVB were in a state of very low heart rate before pacemaker implantation and had no preprocedural 2D-STE, we didn't conduct a comparison of their baseline echocardiography parameters evaluating ventricular synchrony.

In the pacing electrocardiogram, the paced QRS duration of the LBBAP group was significantly shorter than that of the other two groups: LBBAP 113.56 ± 9.66 ms vs. RVA 164.73 ± 14.49 ms, *p* < 0.001; LBBAP 113.56 ± 9.66 ms vs. RVS 148.23 ± 17.3 ms, *p* < 0.001. Meanwhile, the paced QRS duration of the RVS group was significantly shorter than that of the RVA group (148.23 ± 17.3 ms vs. 164.72 ± 14.49 ms, *P* = 0.02). Narrower paced QRS duration may indicate faster biventricular depolarization. Typical examples of surface 12-lead electrocardiograms of the LBBAP, RVA, and RVS groups in our study were shown in Figure 1A respectively. The morphology of the QRS complex was in the form of RBBB in lead V1 for LBBAP. For RVA, the QRS complex presented as a “QS” pattern on leads II, III, AVF, and V1–6, representing the downward direction of ventricular depolarization. The QRS wave morphology of RVS was somewhere in between in terms of width and height.

In 2D-STE, LBBAP resulted in significantly better parameters than the other two groups. Compared with the RVA group, the LBBAP group showed shorter TDmax, SD of the time to peak systolic strain among 18 left ventricular segments, and shorter SPWMD (TDmax, 87.56 ± 56.01 ms vs. 189.85 ± 91.88 ms, *p* = 0.001; SD, 25.40 ± 14.61 ms vs. 67.13 ± 27.40 ms, *p* < 0.001; SPWMD, 28.75 ± 21.89 ms vs. 99.09 ± 46.56 ms, *p* < 0.001). LBBAP also shortened IVMD significantly compared with RVA (−5.38 ± 9.31 ms vs. 44.82 ± 16.42 ms, *p* < 0.001). Similarly, the LBBAP group had shorter TDmax (87.56 ± 56.01 ms vs. 156.46 ± 55.54 ms, *p* = 0.003), SD of the time to peak systolic strain among 18 left ventricular segments (25.40 ± 14.61 ms vs. 49.02 ± 17.85 ms, *p* = 0.001), and SPWMD (28.75 ± 21.89 ms vs. 91.54 ± 26.67 ms, *p* < 0.001) compared with the RVS group. The IVMD was also shorter in the LBBAP group compared with the RVS group (−5.38 ± 9.31 ms vs. 25.31 ± 21.36 ms, *p* < 0.001). When comparing the RVA group with the RVS group, only IVMD was significantly shorter in the RVS group (44.82 ± 16.42 ms vs. 25.31 ± 21.36 ms, *p* = 0.022), with no significant differences observed in characteristics representing intro left ventricular synchrony (Table 2). Those results of 2D-STE indicated that LBBAP contributed to better intro-left ventricular and bi-ventricular contraction synchronization. Figure 1B showed the time-systolic strain curves of the 4CH view, APLAX view, and 2CH view, and the longitudinal strain bull's eye plot for LBBAP vs. RVA vs. RVS, respectively.

**Table 2:** Characteristics evaluating electromechanical synchrony.

	LBBAP (N1 = 16)	RVA (N2 = 11)	RVS (N3 = 13)	*P* value (LBBAP vs RVA)	*P* value (LBBAP vs RVS)	*P* value (RVA vs RVS)
Electrocardiogram						
Paced QRS duration, ms	113.56 ± 9.66	164.73 ± 14.49	148.23 ± 17.3	<0.001	<0.001	0.02
Echocardiography						
SPWMD, ms	28.75 ± 21.89	99.09 ± 46.56	91.54 ± 26.67	<0.001	<0.001	0.624
TDmax, ms	87.56 ± 56.01	189.85 ± 91.88	156.46 ± 55.54	0.001	0.003	0.285
SD, ms	25.40 ± 14.61	67.13 ± 27.40	49.02 ± 17.85	<0.001	0.001	0.064
IVMD, ms	−5.38 ± 9.31	44.82 ± 16.42	25.31 ± 21.36	<0.001	<0.001	0.022
LVEF’, %	66.19 ± 6.29	62.36 ± 5.24	64.08 ± 4.63	0.110	0.323	0.404

SPWMD, the time of septal-to-posterior wall motion delay; TDmax, the maximum difference of the time to peak systolic strain among the 18 left ventricular segments; SD, the standard deviation of the time to peak systolic strain among the 18 left ventricular segments; IVMD, interventricular mechanical delay; LVEF’, left ventricular ejection fraction after pacemaker implantation.

### Atrial high-rate episodes

**Figure 3:**
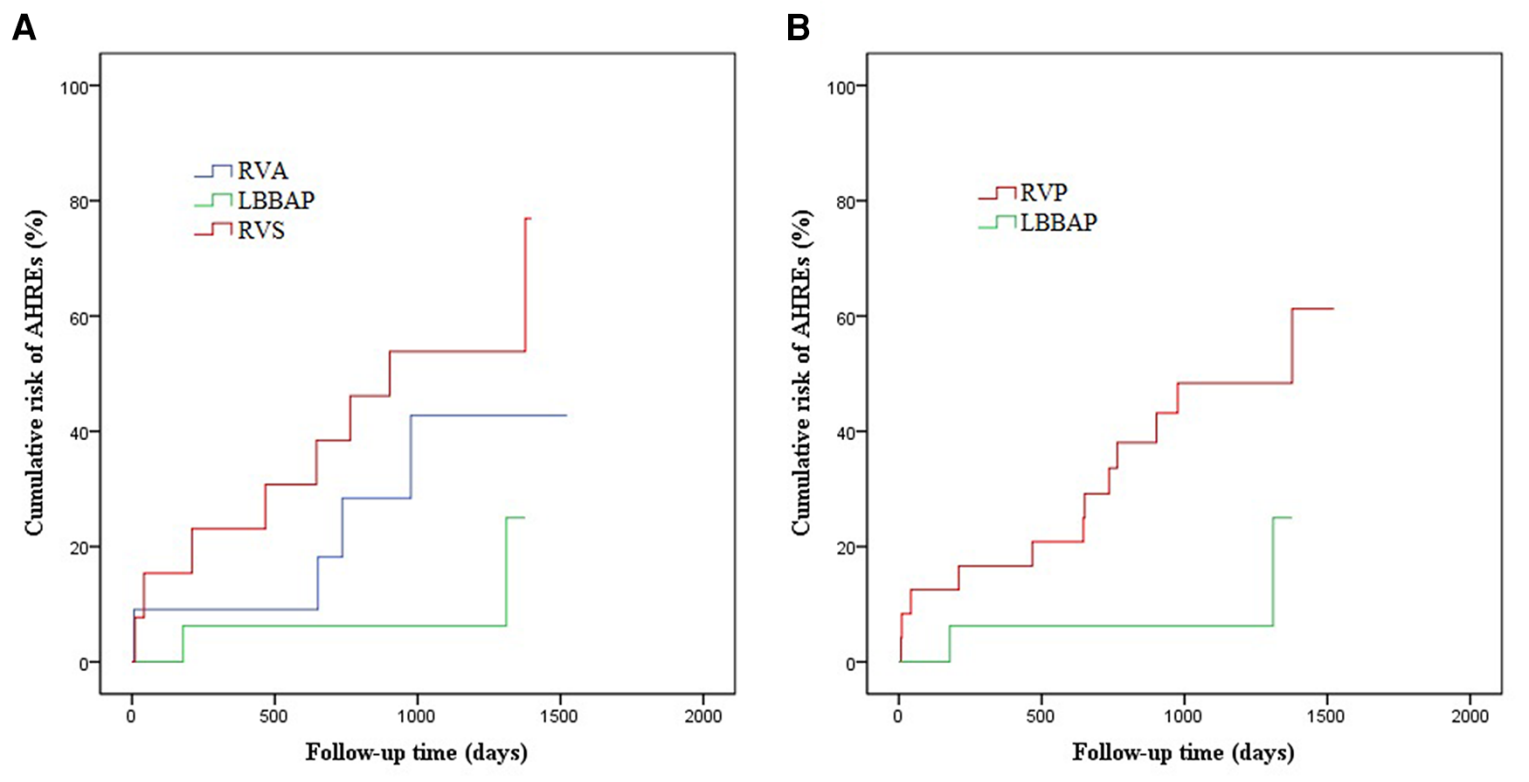
The Kaplan–Meier curve for cumulative risk of AHREs in patients with LBBAP vs RVP vs RVS. (**A**) LBBAP vs RVA, log-rank *p* = 0.139; LBBAP vs RVS, log-rank *p* = 0.027; RVA vs RVS, log-rank *p* = 0.312; (**B**) LBBAP vs RVP, log-rank *p* = 0.043.

During follow-up, novel AHREs were detected in a total of 14/40 (35%) patients, with occurrences in 2/16 (12.5%) from the LBBAP group, 4/11 (36.4%) from the RVA group, and 8/13 (61.5%) from the RVS group. Figure 3 illustrates the Kaplan-Meier (KM) curves for the cumulative risk of AHREs stratified by pacing sites. The incidence of AHREs in the LBBAP group was significantly lower than in the RVS group (log-rank *p* = 0.027). But there was no significant difference between the RVA group and the RVS group, or between the LBBAP group and the RVA group. When combined RVA group and RVS group as RVP group, LBBAP was proved to be independently associated with decreased risk of AHREs compared with RVP (log-rank *p* = 0.043). According to the results of the last echocardiography examination during the follow-up, no severe valvular stenosis or regurgitation was found in the three groups. Only 2 patients in the RVA had mild to moderate tricuspid regurgitation, and 5 patients in the RVS group had mild to moderate tricuspid regurgitation.

## Discussion

The present study analyzed AVB patients with LBBAP and RVP (including RVA and RVS), and the major findings were as follows: (i) LBBAP was associated with a more physiological ventricular activation evidenced by a narrower paced QRS duration and better intro-left ventricular and bi-ventricular mechanical synchrony than either RVA or RVS; (ii) Compared with RVA, RVS improved bi-ventricular synchrony and made narrower QRS, but no significant difference in intro-left ventricular synchrony was found; (iii) LBBAP was independently associated with a decreased risk of AHREs compared with RVP.

Although traditional RVP is well tolerated in patients with normal cardiac function, the ventricular dyssynchrony generated by RVP is found to be associated with an increased risk of heart failure, cardiomyopathy, and arrhythmias, especially in patients with high ventricular pacing burden (2, 5). Huang W et al. (8) developed a novel pacing strategy, the left bundle branch area pacing (LBBAP), in 2017. As far as we know, the LBB conduction fibers have a large distribution range under the endocardium of the ventricular septum. For LBBAP, the lead is implanted slightly distal to the AV block area and screwed through the septum to capture the left bundle branch and activate the Purkinje conduction system in sequence. In that way, the entire ventricle is electrically activated as physiologically as possible. Recently, Marek Jastrzębski et al. (16) analyzed 2,533 patients with LBBAP coming from 14 European centers, and they found that LBBAP is feasible as a primary pacing technique for bradyarrhythmia.

Theoretically, LBBAP might overcome the adverse effects caused by dyssynchrony due to RVP. Zhu H et al. (17) reported that LBBAP significantly reduced the risk of new-onset AF compared with RVP (HR 0.199, 95% CI:0.105–0.378, *P* < 0.001) in patients with ventricular pacing percentages ≥20%. Venkatesh Rav et al. (18) also found that patients with high ventricular pacing burden were more likely to benefit from LBBAP with a lower risk of developing AF. In this study, we evaluated the differences between LBBAP and traditional RVP in the development of AHREs and demonstrated a lower incidence of AHREs with LBBAP. AHREs are considered to be manifestations of atrial electrical remodeling, which is characteristic of atrial cardiomyopathy (19). To some extent, AHREs are considered a precursor to further atrial arrhythmias such as AF (20–22). In the study by Jeff S Healey et al, they found that AHREs occurred frequently in patients with pacemakers (10.1% by 3 months), and AHREs were proved to be associated with increased risk of clinical AF (HR 5.56, 95% CI: 3.78–8.17, *P* < 0.001) and thromboembolism (HR 2.49, 95% CI:1.28–4.85, *P* = 0.007) (23).

The mechanism of the increased risk of AHREs associated with ventricular pacing is unclear. The left atrial dysfunction that resulted from left ventricular dyssynchrony may be an important explanation. The main functions of the left atrium are closely related to left ventricle mechanics: reservoir (storing blood returning from the pulmonary veins during left ventricular systole), conduit (conducting the pressure of the pulmonary vein and left ventricle), and booster pump (active contraction pumping blood into the left ventricle at the end of left ventricular diastole) (24). Thriveni Sanagala et al. (25) found that RVP acutely impaired left atrium emptying and increased its minimal volume. It was also demonstrated that reduced left atrial function markedly increased the propensity for new-onset atrial arrhythmia, independent of other clinical risk factors (26, 27). Binni Cai et al. (28) reported that the left ventricle synchrony in the LBBAP group was significantly superior to that in the RVP group. Our study analyzed the results of ECG and 2D-STE examinations and demonstrated that LBBAP can achieve better intro-left ventricular contraction synchronization and lower the risk of AHREs than RVP. What's more, RVS failed to improve left ventricular systolic synchrony, although it shortened IVMD and generated a narrower QRS compared with RVA, and ultimately failed to obtain a significant difference in affecting the risk of AHREs. Hence, we hypothesized that the physiological electromechanical activation of the left ventricle generated by LBBAP could potentially minimize impairment of left atrial function and consequently reduce the risk of AHREs.

Analyzing ventricular electromechanical synchrony through non-invasive examination has always been a challenge. In recent years, 2D-STE has been utilized in a number of clinical scenarios to quantify ventricular synchrony. However, left ventricular mechanics are inherently three-dimensional, characterized by complex spatial orientation and simultaneous contraction in diverse directions. Recognizing these complexities, three-dimensional speckle tracking echocardiography (3D-STE) has emerged as a more precise tool for assessing left ventricular mechanics (29). Nonetheless, 3D-STE typically necessitates patient breath-holding and stable rhythm to facilitate ECG-gated multi-beat 3D acquisition. Moreover, the metrics, reference values, and cutoffs for 3D strain parameters currently vary among vendors and are heavily reliant on the specific 3D ultrasound equipment used. Given these considerations, including constraints related to ultrasound equipment and financial issues, 3D-STE was not used in our study. Further studies using 3D-STE are expected to provide a more comprehensive analysis.

Although the LBBAP technique is very promising, there is still concern regarding its long-term effectiveness and safety. Unipolar pacing in LBBAP might selectively capture LBB and rapidly transmit the electrical impulses along the cardiac conduction system, but bipolar pacing might compensate for right ventricular (RV) delay by anodal capture of RV septum and RV preexcitation (11). In addition, bipolar pacing is less susceptible to interference and generating superior sensing and pacing safety (7). Therefore, bipolar pacing at an acceptable pacing output (3.5 V/0.42 ms) was utilized in our study. This choice aligns with our main research objective.

LBBAP cannot overcome the problem of intraventricular block, and these patients might not achieve additional benefits from LBBAP (14). Currently, physiological conduction system pacing is recommended in patients with cardiac insufficiency or patients highly dependent on pacemakers to reduce the risk of pacing cardiomyopathy (7). Our results suggested that patients with normal cardiac function requiring high percentage pacing might also benefit from LBBAP by increasing ventricular synchrony and decreasing the risk of incident AHREs.

## Limitation

As a retrospective observational study, several limitations should be considered when interpreting the results. Patients with known clinical AF were excluded, but patients with asymptomatic AF may be missed. Patients with known cardiomyopathy and those identified with cardiomyopathy on preoperative routine echocardiography were excluded, but patients in a compensatory phase who haven't yet exhibited abnormal ventricular wall motion or reduced ejection fraction may be missed. Performing a preoperative 2D-STE examination may offer an opportunity to detect these patients earlier. During follow-up, the treatment strategy and conditions of comorbidities were not systematically reviewed, preventing us from adjusting for their potential impact on the incidence of AHRE events. Due to the limited number of patients examined by 2D-STE, the sample size of this study is small. The small sample size also restricted this study from performing multivariable analysis on AHRE events. These findings should be further validated in randomized studies with a larger sample size. Our study underscores the necessity for further investigations utilizing clinical non-invasive examinations to deepen our understanding of these pacing modalities.

## Conclusions

Our study outlines that LBBAP offers advantages in terms of narrower-paced QRS duration, improved intro-left ventricular and biventricular contraction synchronization, and a decreased risk of AHREs compared with traditional RVP. These findings suggest the potential clinical benefits of LBBAP in improving cardiac function and reducing the risk of arrhythmias in AVB patients with high ventricular pacing burden. Further research and larger-scale studies are warranted to validate these observations and explore their impact on long-term clinical outcomes.

## Data Availability

The datasets presented in this article are not readily available because The raw/processed data required to reproduce these findings cannot be shared at this time as the data also forms part of an ongoing study. Requests to access the datasets should be directed to Z-JS, szj1979@126.com.
